# 414. Sex differences in clinical presentation and outcomes amongst hospitalized patients with COVID-19 in Singapore

**DOI:** 10.1093/ofid/ofad500.484

**Published:** 2023-11-27

**Authors:** Matthew C Y Koh, Jinghao Nicholas Ngiam, Srishti Chhabra, Wilson Goh, Meng Ying Sim, Nicholas W S Chew, Ching-Hui Sia, Gail Cross, Paul Tambyah

**Affiliations:** National University Health System, Singapore, Singapore; National University Health System, Singapore, Singapore; National University Health System, Singapore, Singapore; National University Health System, Singapore, Singapore; National University Health System, Singapore, Singapore; National University Heart Centre, Singapore, Not Applicable, Singapore; National University Heart Centre, Singapore, Not Applicable, Singapore; National University Health System, Singapore, Singapore; National University Hospital, Singapore, Singapore, Not Applicable, Singapore

## Abstract

**Background:**

Sex differences in clinical presentation and outcomes in patients with coronavirus disease 2019 (COVID-19) remain to be clearly defined. We sought to evaluate sex differences in the presentation and outcomes of COVID-19 in our Asian context.

**Methods:**

We retrospectively examined 1781 consecutive patients hospitalised from 2020 to 2021, with polymerase chain reaction confirmed SARS-CoV-2 infection. Patients were divided into males and females. Baseline clinical characteristics, laboratory findings as well as clinical outcomes were compared. Adverse composite clinical outcomes were defined as requiring supplemental oxygenation, intensive care or mortality.

**Results:**

Of the 1781 patients studied, 351 (19.7%) were female. The male predominance in our cohort were due to large outbreaks of COVID-19 in dormitory workers early in the pandemic. Female patients had more medical comorbidities, and were slightly older (46.4±18.3 vs 41.1±13.4 years, p< 0.001). They had a lower serum ferritin level (174.6±336.7 vs 216.7±284.7 µg/L, p=0.027), but no significant difference in the serum C-reactive protein (16.9±29.1 vs 14.4±29.8 mg/dL, p=0.187). Females were more likely to require supplemental oxygenation (7.1% vs 2.7%, p=0.025) and intensive care (4.8% vs 2.7%, p=0.034), but we did not demonstrate a difference in mortality (0.9% vs 0.7%, p=0.728). On multivariable analysis, female gender remained independently associated with adverse composite outcomes (adjusted OR 1.89, 95%CI 1.17-3.04) after adjusted for dormitory exposure and prior vaccination against COVID-19.

Comparing COVID-19 clinical presentation and outcomes in females versus males amongst hospitalised patients from 2020 to 2021

**Figure:**
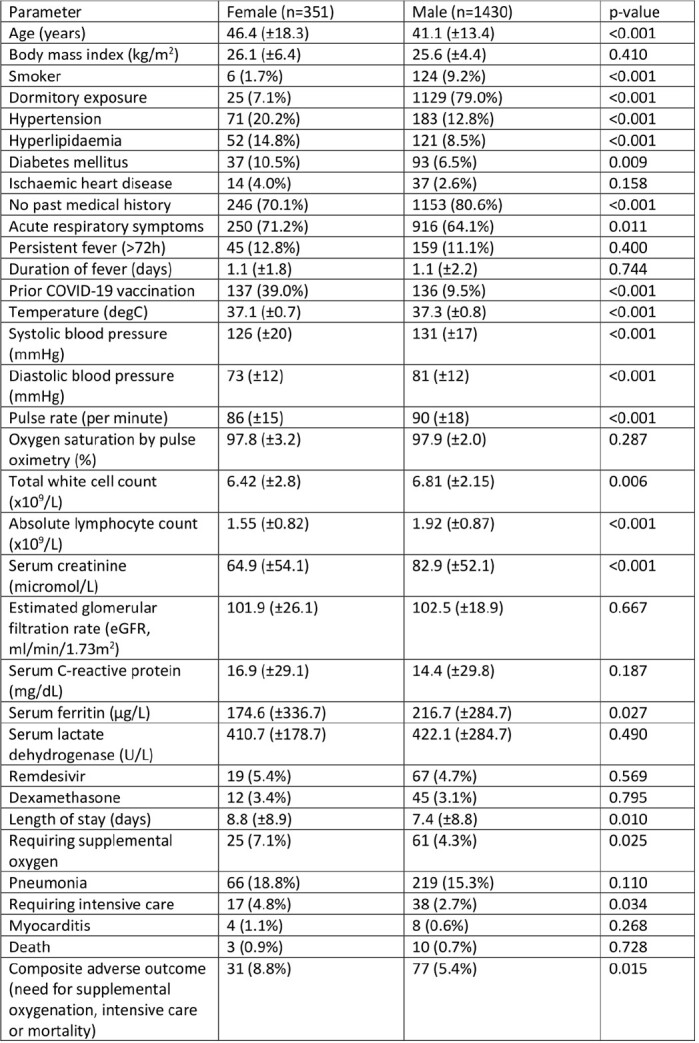

Multivariable analysis showing female gender independently associated with increased odds of adverse clinical outcomes in COVID-19 illness

**Figure:**
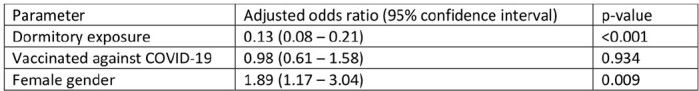

**Conclusion:**

Female sex may be associated with adverse clinical outcomes in hospitalised patients with COVID-19. Further study is needed to elucidate and investigate the pathophysiological mechanisms behind this observation.

**Disclosures:**

**All Authors**: No reported disclosures

